# NSAIDs between past and present; a long journey towards an ideal COX-2 inhibitor lead

**DOI:** 10.1039/d4ra04686b

**Published:** 2024-09-25

**Authors:** Nadia A. Khalil, Eman M. Ahmed, Toka Tharwat, Zeinab Mahmoud

**Affiliations:** a Pharmaceutical Organic Chemistry Department, Faculty of Pharmacy, Cairo University 33 Kasr El-Aini Street Cairo 11562 Egypt zeinab.mahmoud@pharma.cu.edu.eg

## Abstract

Nonsteroidal anti-inflammatory drugs (NSAIDs) are the most abundantly used classes among therapeutic agents in medicine. NSAIDs inhibit the enzyme cyclooxygenase (COX), which is responsible for the conversion of arachidonic acid to prostaglandins. Meanwhile, non-selective NSAIDs are considered as a double-edged weapon since inhibition of COX-1 can lead to gastrointestinal side effects and kidney damage, whereas selective COX-2 inhibition provides anti-inflammatory effects without gastrointestinal toxicity. The detection of COX-2 role in inflammation process launched a new era in its management. Several trials have been established to proceed towards selectivity of well-defined anti-inflammatory members. COX-2 selective inhibitors are evidently safer on the gastrointestinal tract than non-selective NSAIDs. Nevertheless, their unexpected cardiovascular risks cannot be ignored. This review article highlights the latest trials aimed at developing new compounds with promising selective COX-2 activity.

## Abstract 2

Nonsteroidal anti-inflammatory drugs (NSAIDs) are the most abundantly used classes among therapeutic agents in medicine.

## Graphical abstract

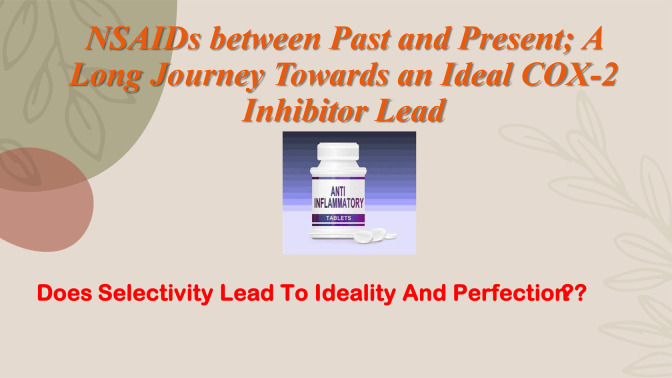

## 1. Introduction

Inflammation is a normal, essential immune system response through diverse interactions between soluble factors and cells. Inflammation occurs in any tissue during exposure to infections, toxins, or autoimmune disease processes.^1^ From a pathological point of view, inflammation can be categorized into acute and chronic inflammation. Acute inflammation has a fast onset and a short duration of time with several symptoms such as pain, redness, heat, swelling and loss of function in severe cases.^2^ On the other hand, chronic inflammation exhibits slower onset of symptoms while persisting for a longer duration of time.^3,4^ Normally, this process contributes to the recovery from infection and consequently healing. However, if selective degradation and aided reconstruction of the inflamed cells are inadequately phased out, this can lead to a persistent tissue damage by leukocytes, lymphocytes.^5,6^

**Fig. 1:**
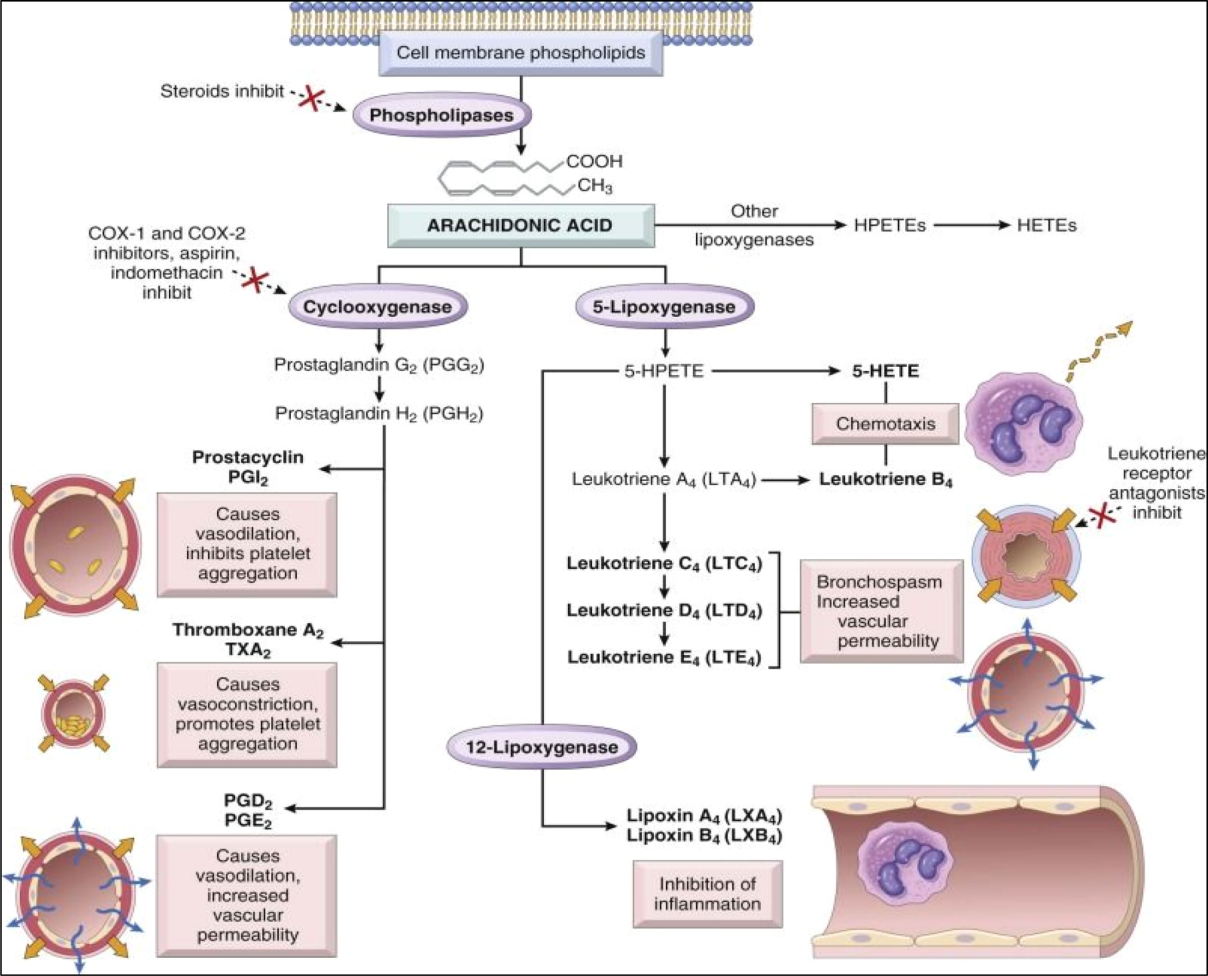
Arachidonic acid metabolism products involved in the inflammatory process.^7^

The normal pathway of inflammation starts with trauma, infection, or immune reactions. Such an incidence stimulates the cleavage of membrane's phospholipids by phospholipase A_2_ enzyme to produce arachidonic acid. In accordance, arachidonic acid is subjected to downstream modification with cyclooxygenase enzymes into prostaglandins G_2_ (PG_2_) and then converted to PGH_2_. PGH_2_ is a precursor that can be further converted into various prostaglandins, thromboxanes, and other eicosanoids (Fig. 1).^6,8–10^ Prostaglandins (PGs) are hormone-like bioactive substances that are directly implicated in many physiological and pathological processes.^10^ PGs are considered to be significant mediators involved in various therapeutic areas of concern, including inflammation, pain, pyrexia, cancer, glaucoma, male sexual dysfunction, osteoporosis.^8^ On the other hand, thromboxane A_2_ (TXA_2_) possesses vasoconstrictor and platelet aggregation effect.^8^ Meanwhile, 5-lipoxygenase enzyme (5-LOX) acts on arachidonic acid to produce leukotrienes (LTs). Leukotrienes (LTs) are responsible for anaphylaxis. Accordingly, therapies inhibiting inflammatory mediators are considered as an effective treatment for controlling inflammation.^11–14^

Depending on the enzyme targeted in the inflammation process, we can classify the anti-inflammatory agents into two major classes, steroidal anti-inflammatory agents, and non-steroidal anti-inflammatory ones.

### 1.1 Steroidal anti-inflammatory agents (glucocorticoids)

**Fig. 2:**
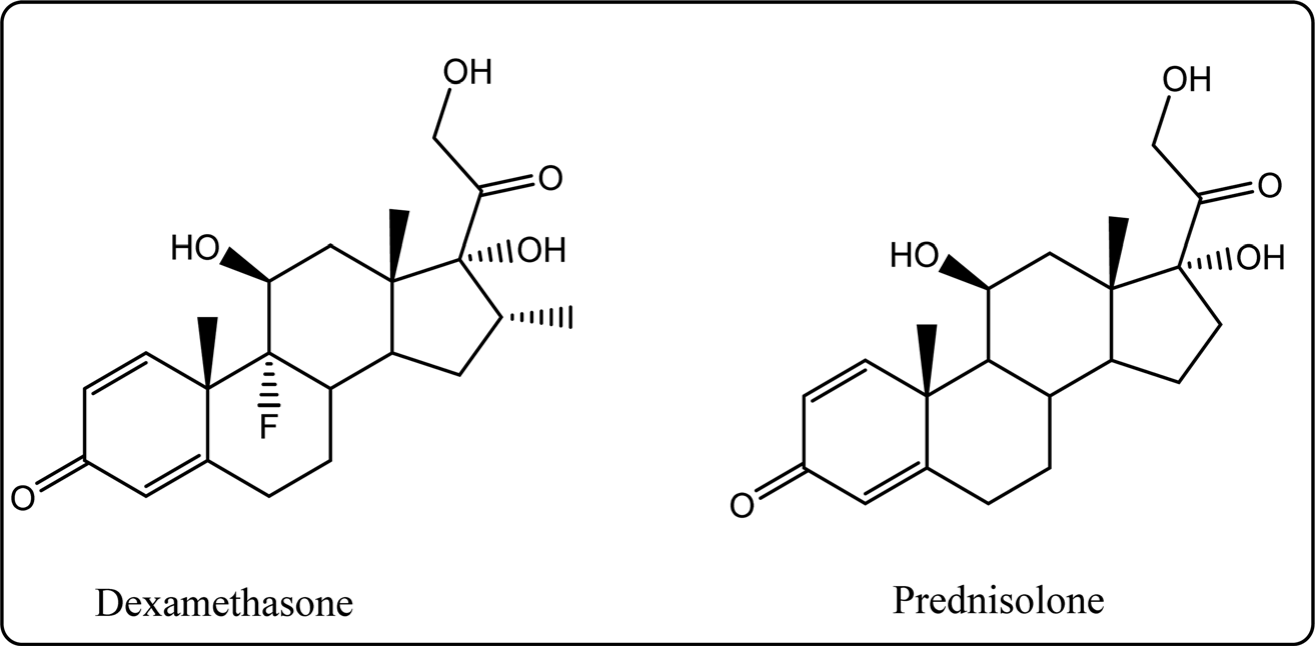
Structures of the most famous corticosteroids.

Glucocorticoids (GC) stop inflammation pathway by inhibiting phospholipase A_2_ (PLA_2_), which consequently reduces the production of arachidonic acid.^15^ The major use of such a class is mostly for patients suffering from diseases such as rheumatoid arthritis, osteoarthritis.^16,17^ Although GCs can be considered as a potent drug, their excessive use specifically in large doses for a long duration of time is associated with overwhelming dermatologic, musculoskeletal side effects. Suppression of the hypothalamic pituitary adrenal gland (HPA) axis or cushing syndrome are other expected possible drawbacks. Moreover, the gastrointestinal, ocular, cardiovascular, neuropsychiatric, and immunologic are serious side effects for glucocorticoids long-term use^15,16,18^ (Fig. 2).

### 1.2 Non-steroidal anti-inflammatory drugs (NSAIDs)

NSAIDs is a class of pharmaceutical agents most generally used to manage pain, fever, and numerous inflammatory forms. It is worth mentioning that there are two main COX enzymes possessing different functions, COX-1, and COX-2. COX-1 is indeed constitutively expressed and plays a role in maintaining homeostasis in the gastrointestinal tract, kidneys, and other organs. On the other hand, although COX-2 is primarily induced during inflammation, it also is constitutively expressed in some tissues and has roles in certain physiological processes such as renal function and healing.^19,20^ However, most of the NSAIDs cause significant gastrointestinal side effects due to COX-1 enzyme suppression^20–22^ while several studies proved that selective inhibition of COX-2 minimizes the GIT effects, and other undesired associated side effects.^21^

**Fig. 3:**
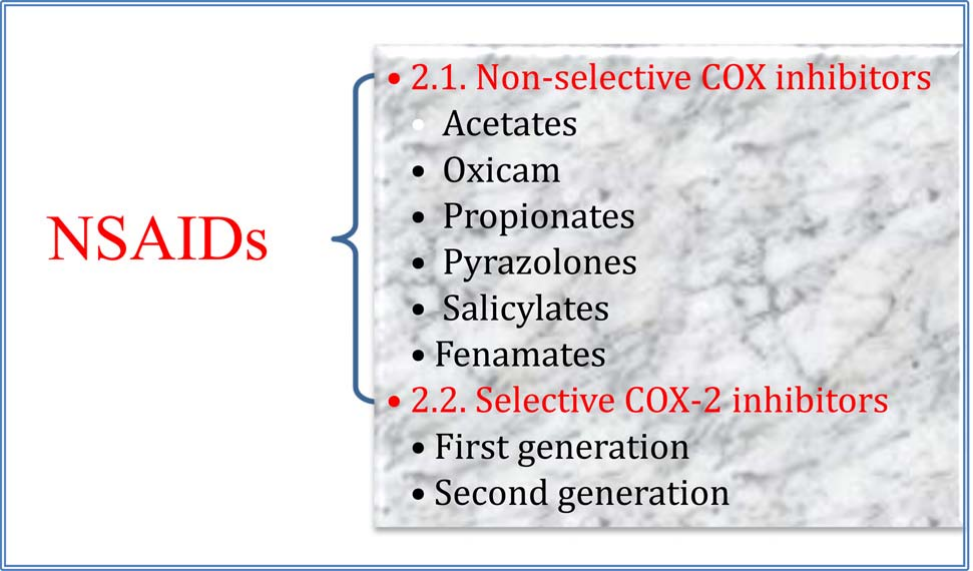
Main classes of NSAIDs.

From the isoenzyme's selectivity point of view, NSAIDS can be categorized into non-selective and selective COX-2 inhibitors (Fig. 3).

#### 1.2.1 Non-selective COX inhibitors

**Fig. 4:**
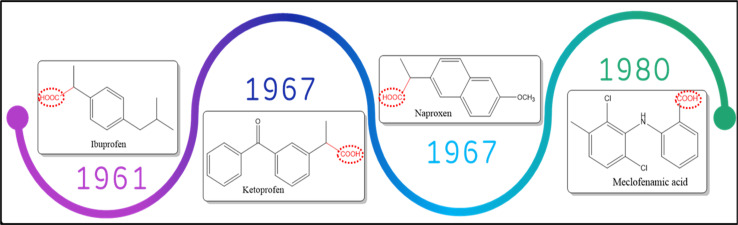
Chronological order for the most famous non-selective COX inhibitors.

This class lacks the selective inhibitory action on COX isoforms with fewer side effects in comparison with GCs class. Famous members for this class are ibuprofen, ketoprofen, naproxen and aspirin ^23^ (Fig. 4). Although this group is still recommended for the use as analgesic and anti-inflammatory agents in rheumatoid arthritis, degenerative joint disease, ankylosing spondylitis and periarticular diseases such as tendinitis,^24^ they cause serious gastrointestinal tract adverse effects. Gastrointestinal tract injury is attributed to two main reasons, the direct insult of the carboxylic acid moiety (–COOH) with GIT mucosal cells in combination with the decrease in PG production in tissues thus reducing the cytoprotective effect of PGs on GIT health.^25,26^

#### 1.2.2 Selective cyclooxygenase-2 enzyme (COX-2) inhibitors (COXIBs)

**Fig. 5:**
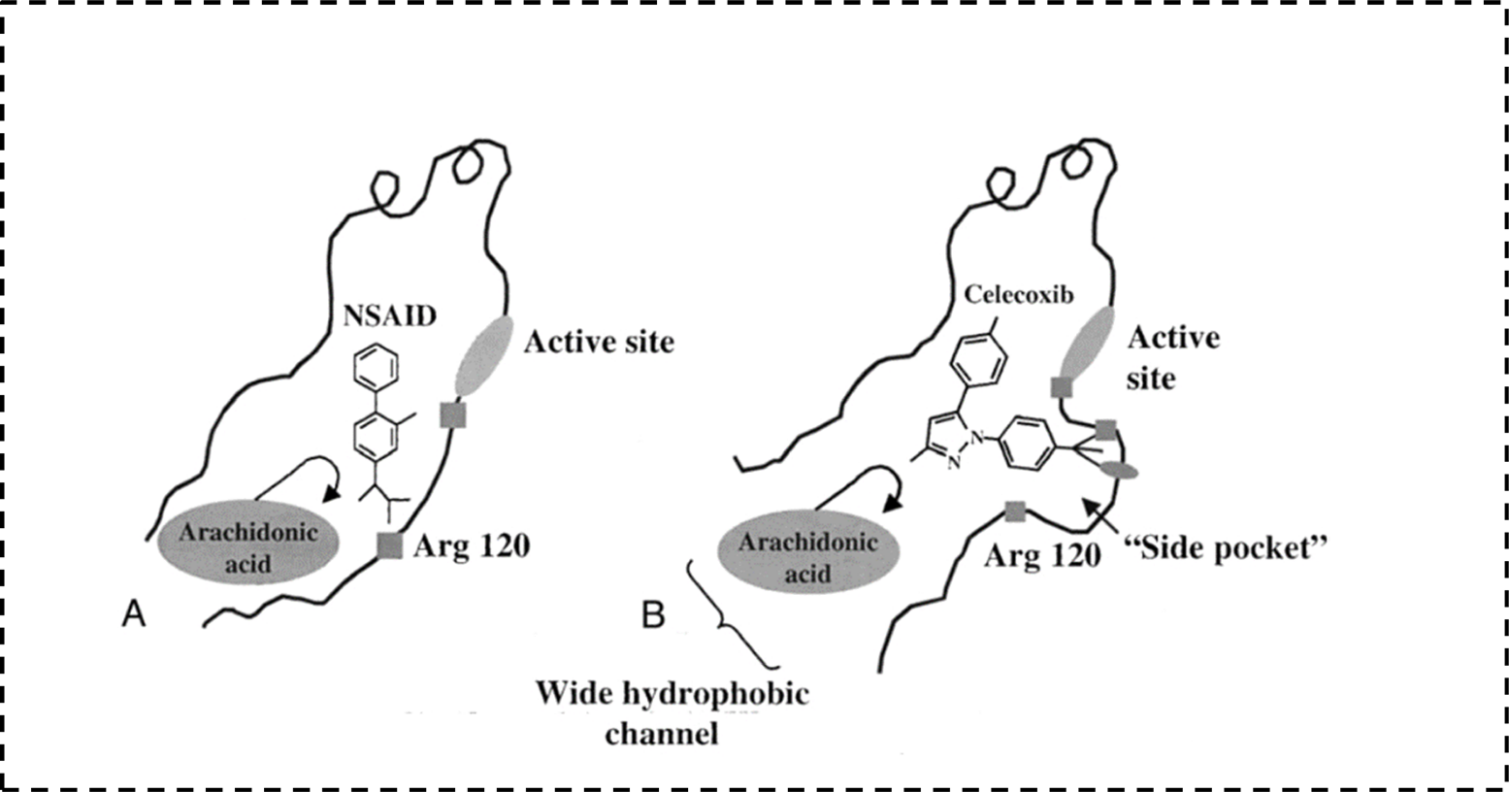
Prominent features distinguishing the COX isoenzymes.^33,34^

The search for ideality is one of the scientists' targets. Accordingly, researchers started to look for another alternatives aiming to avoid GIT side effects caused by non-selective NSAIDs.^23,27^ The first step in this journey was the discovery of COX isoforms. In early 1990s, Dan Simmons *et al.* raised the fact that COX does not exist as an only one enzyme. Actually, there are two isoenzymes of COX with structural and functional differences.^28–31^ From the structural point of view there is a very small but still a significant difference between COX-1 and COX-2 isoenzymes.^32^ Both enzymes have the same cavity to which arachidonic acid fits. On the other hand, the main difference is detected in the presence of a small additional pocket in COX-2 enzyme structure. So the key for increasing selectivity towards COX-2 enzyme is having an extension that specifically binds to COX-2 additional pocket (Fig. 5).^33,35–37^ Moreover, Table 1 highlights the most significant points of differences between the two isoenzymes.

**Table 1:** The most significant points of difference between COX-1 and COX-2 (ref. 34 and 38–40)

Points of comparison	COX-1	COX-2
Active site	NSAIDs generally bind the top part of the COX channel located near Tyr385 and Arg120 which is present at the entrance of the COX channel^34^	
Significant amino acids	(a) Isoleucine 523, (b) Isoleucine 434, Histidine 513	A smaller valine at amino acid residue at position 523, a valine amino acid also at position 434, and the secondary pocket which is accessible at the COX-2 active site
Volume of COX binding site	316 Å (ref. 21)	394 Å (ref. 21)

**Fig. 6:**
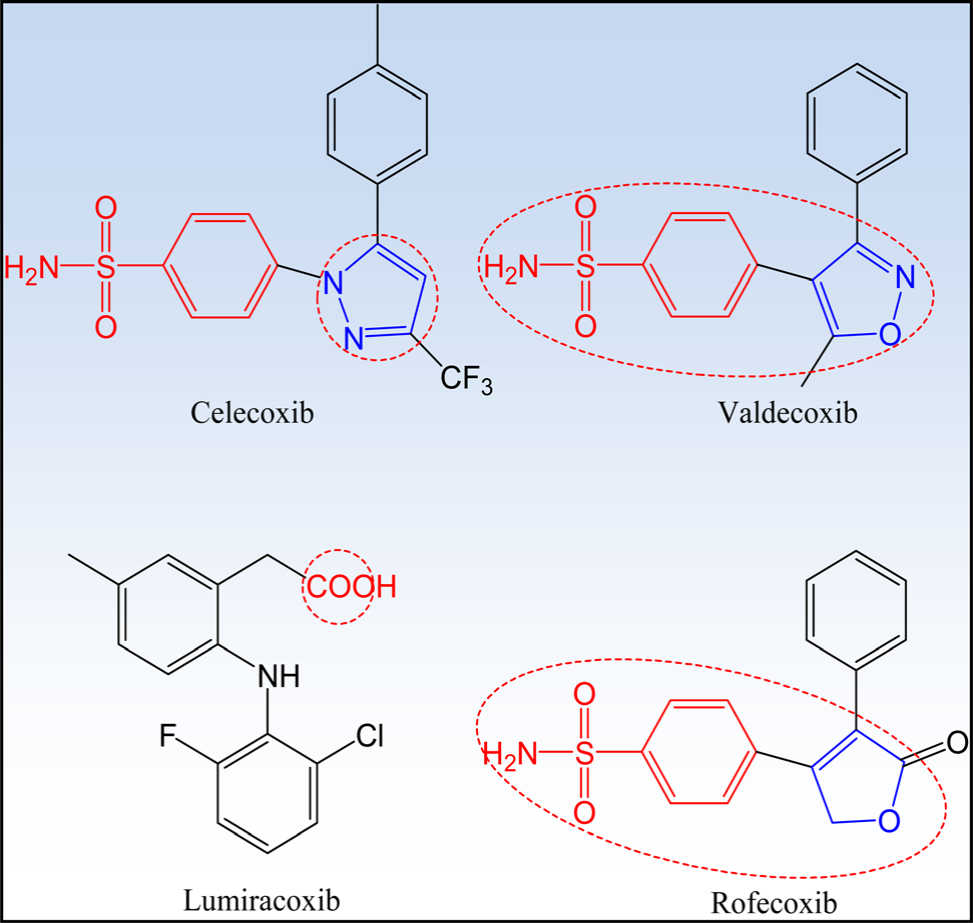
Pharmacophores of selective COX-2 inhibitors.

COXIBs are selective COX-2 inhibitors characterized by the pharmacophore carboxylic or heterocyclic five-membered ring. Structure activity relationship studies proved that substituted sulfonyl groups are also considered as pharmacophores that recognize COX-2 active site pocket^4,21,41,42^ (Fig. 6).

They are used in the management of inflammatory pain, symptoms of osteoarthritis and rheumatoid arthritis with enhanced gastrointestinal safety profile compared to NSAIDs.^43,44^ Although, COXIBs cause lesser ulceration than traditional NSAIDs they can increase the risk of severe cardiovascular events such as heart attack, myocardial infarction (MI), and stroke.^45–47^ The safety of NSAIDs is influenced by multiple factors, not just their selectivity for COX-1 or COX-2. Both the COX-1 and COX-2 roles are inhibited during NSAID therapy.^20,48^

##### 1.2.2.1 First generation COXIBs

**Fig. 7:**
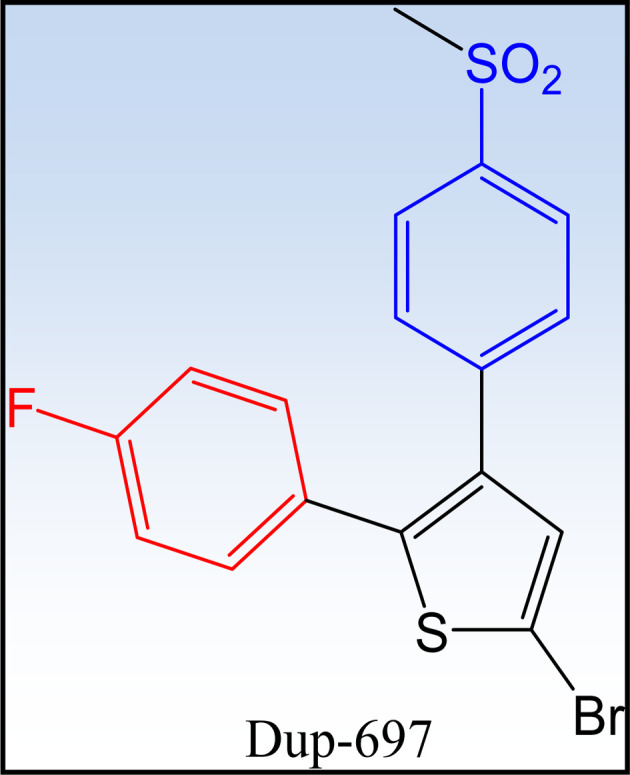
DuP-697 is one of the lead compounds in the development of COX-2 inhibitors.

Celecoxib® and rofecoxib® are two earliest and most widely recognized COX-2 inhibitors (Fig. 6). DuP-697 is one of the lead compounds that inspired the development of selective COX-2 inhibitors (Fig. 7). The comparison between the two drugs is illustrated in Table 2.^55–57^

**Table 2:** Celecoxib and rofecoxib main differences^4,29,49^

	Celecoxib	Rofecoxib
History	The first approved selective COX-2 inhibitor. It was marketed by Pfizer	A selective COX-2 inhibitor produced by Merck^50,51^
Trade name	Celebrex®, and elyxyb®	Vioxx® (ref. 52)
Ratio of COX-2/COX-1 inhibition	7.6-Fold	35-Fold
Adverse side effects	Dyspepsia, edema, moderate probability of CVS side effects^53^	
SAR	Both members possess a diaryl substitution on a central heterocycle^54^	

An acceptable explanation for the cardiovascular undesired side effects is the inhibition of endothelial PGI_2_ synthesis, which is mainly dependent on COX-2. PGI_2_ is the powerful inhibitor of platelet aggregation and thrombosis. Consequently, affecting PGI_2_ synthesis leads to triggering the acute coronary syndromes, myocardial infarction, thrombosis and atherosclerosis are expected.^58,59^

##### 1.2.2.2 Second generation COXIBs

**Fig. 8:**
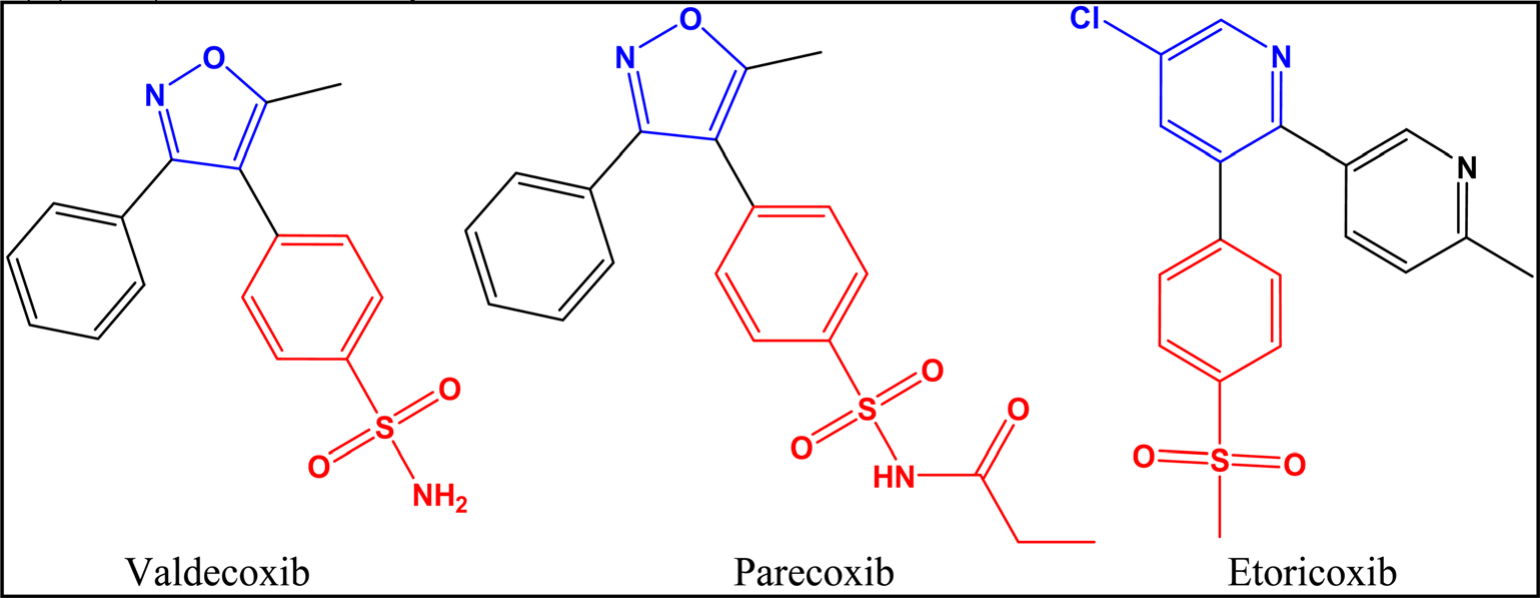
Second generation of COXIBs.

The most important members of this class are valdecoxib, parecoxib and etoricoxib (Fig. 8).^30,47^ A brief description for the characteristics of each member is illustrated in Table 3.

**Table 3:** Characteristics of the members of COXIBs second generation^4,29,47,60^

	Valdecoxib	Parecoxib	Etoricoxib
History	FDA-approved valdecoxib in 2001, shortly it was withdrawn by Pfizer at 2005 due to it’s cardiovascular and severe skin reaction risks	Patency was in 1996 and FDA-approved in 2002, then produced by Pfizer	Discovered and produced by Merck in 2002 and approved for medical use in 2007
Trade names	Bextra®	Dynastat®	Arcoxia®
COX-2/COX-1 inhibition ratio	30-Fold	Water soluble prodrug of valdecoxib	106-fold
Adverse effects	All showed high probability of cardiovascular side effect compared to non-selective NSAIDs^45^		
Central nucleus	Oxazole ring	Oxazole ring	Pyridine ring
SAR	Valdecoxib possesses a diaryl substitution on the central oxazole ring with a characteristic sulfonamide group on one of the aryl rings	Acting as a prodrug, parecoxib possesses the same SAR of valdecoxib	Etoricoxib possesses a diaryl substitution on the central pyridine ring with a characteristic methylsulfonyl moiety on one of the aryl rings

## 2. Promising approaches towards ideal COXIB lead

**Fig. 9:**
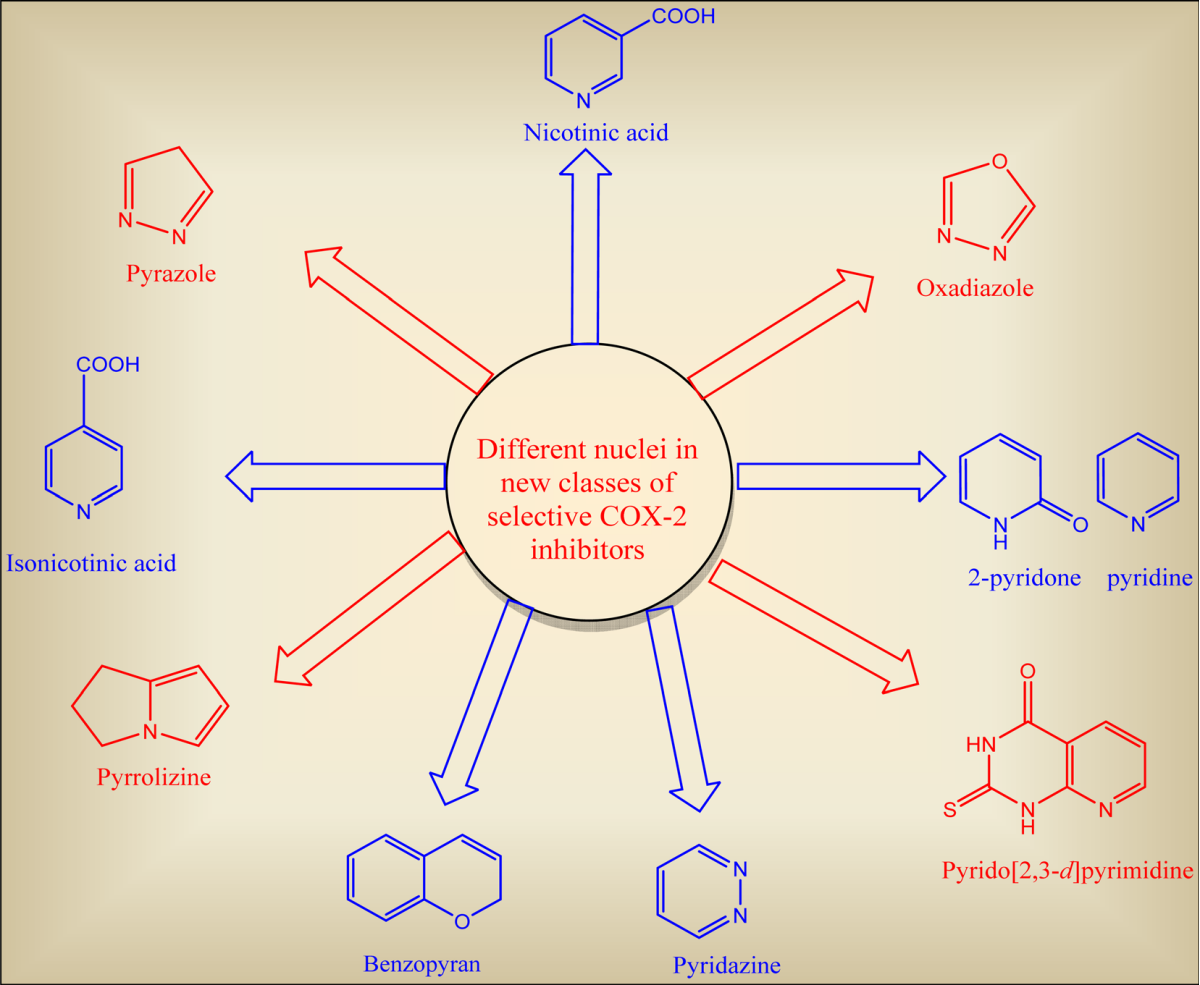
Different heterocyclic nuclei incorporated in selective COX-2 inhibitors scaffold.

Tremendous efforts have been recorded to discover selective COX-2 inhibitors with lesser side effects. The synthesized are categorized according to their common nucleus in each class (Fig. 9).^37,59,61,62^

### 2.1 Nicotinic acid-containing compounds

**Fig. 10:**
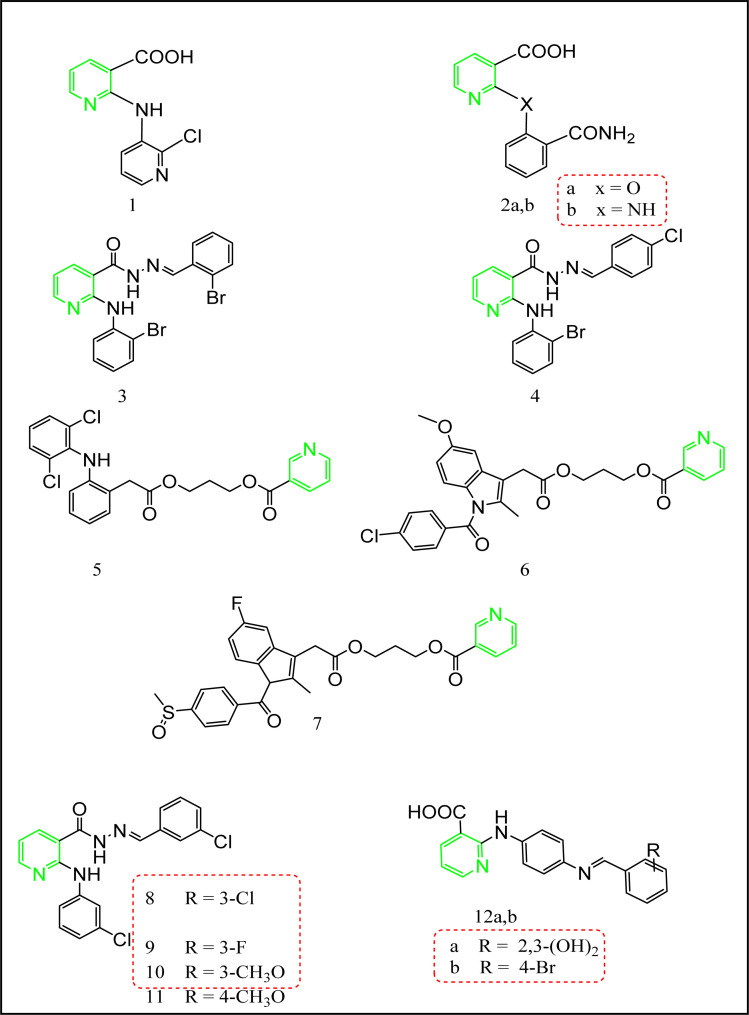
The most promising nicotinic acid-containing compounds.

Abouzid's team synthesized the 2-substituted phenyl derivatives of nicotinic acid 1 and 2a,b (Fig. 10) according to the reported procedure.^12^ These derivatives were biologically evaluated for their anti-inflammatory activity. Compounds 1, 2a,b (Fig. 10) showed unique analgesic and anti-inflammatory activities compared to mefenamic acid as a standard drug. Further investigation concerning the ulcerogenic effect of these compounds were performed.^12^ The screening of all the synthesized compounds revealed that compounds 2a,b exceeded mefenamic acid (54.5 ± 3.45%) regarding anti-inflammatory activity (64.5 ± 4.9%, 61.3 ± 5.6%, and 56.2 ± 5.7%, respectively) with a slightly improved ulcerogenic profile compared to mefenamic acid (ulcer indices = 0.9 ± 0.04%, 0.9 ± 0.03%, and 1 ± 0.08%, respectively *c.f.* 1.1 ± 0.03% for mefenamic acid) and acceptable analgesic activity.^12^ Their tumor necrosis factor TNF-α values were (3.25 ± 0.21%, 2.45 ± 0.18% and 2.67 ± 0.15%, sequentially) and interleukin IL-6 values were (9.2 ± 0.61, 14.3 ± 0.56, and 10.1 ± 0.82 ng mL^−1^).^12^

On the other hand, khalil's research team^12^ succeeded in synthesizing another series of nicotinic acid derivatives (compounds 3 and 4Fig. 10). In accordance, this series was prepared *via* the straightforward nucleophilic substitution reaction of the respective primary aromatic amines with the 2-chloronicotinic acid. The activation of the carboxylic functionality was achieved through esterification followed by the reaction with hydrazine hydrate to afford the hydrazide derivatives. Finally, compounds 3 and 4 were synthesized through the condensation reaction of the respective aldehydes with hydrazide derivatives. Applying carrageenan induced rat paw edema,^63^ compounds 3 and 4 showed superior anti-inflammatory activity percentage of edema inhibition = 52.9 ± 3.8%, and 62.2 ± 5.4%, respectively *c.f.* mefenamic acid percentage of edema inhibition = 59.3 ± 4.21%), also they exhibited unique analgesic activities (61.7 ± 4.8%, and 61.7 ± 4.8%, successively *c.f.* mefenamic acid analgesic activity 72.4 ± 4.6), and an enhanced ulcerogenic profile (0.82 ± 0.05%, and 1.3 ± 0.09%, sequentially).^64^

**Fig. 11:**
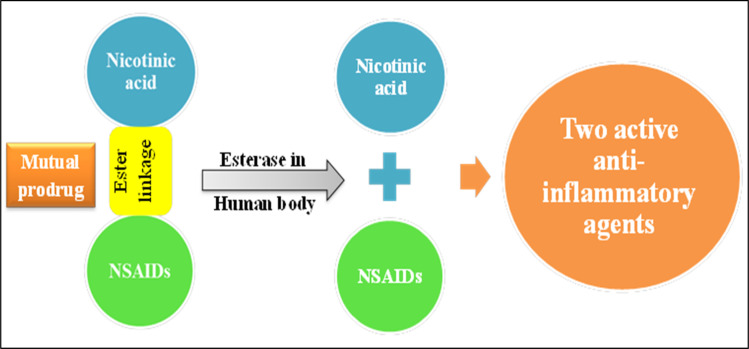
Mutual prodrugs strategy.

Furthermore, new scaffolds of nicotinic acid derivatives 5, 6, and 7 (Fig. 11) were reported by Gund *et al.*^65^ The aim of this study was to combine a well-known NSAID with the nicotinic acid to benefit from the mutual prodrug strategy of different therapeutic agents in a single molecule by adopting the 1,3-propandiol ester linkage (Fig. 11).^65,66^ The 1,3-propandiol ester linkage was adopted for such an aim.

These conjugates 5, 6 and 7 are privileged in the extended time of action, and as a result, decreasing the number of doses. Regarding their *in vitro* anti-inflammatory activity, the human TNF-α and IL-6 parameters were assessed. All the synthesized scaffolds have considerable *in vitro* potency when evaluated with the corresponding parent drug. Compound 5 as a hybrid of nicotinic acid and diclofenac showed IL-6 and TNF-α inhibition activities (44 ± 4.1%, and 48 ± 1.9%, respectively), on the other hand diclofenac itself showed IL-6 and TNF-α inhibition activities (35 ± 3.2%, and 42 ± 3.2%, respectively). Furthermore, compound 6 compared to indomethacin as a standard drug revealed IL-6 and TNF-α inhibition activities (37 ± 3.9%, and 40 ± 2.1%, sequentially), in contrast to indomethacin IL-6 and TNF-α inhibition activities (21 ± 3.5%, and 32 ± 7.2%, respectively). Compound 7 exceeded sulindac IL-6 and TNF-α inhibition activities (42 ± 3.1%, and 44 ± 7.1%, respectively), *c.f.* sulindac IL-6 and TNF-α inhibition activities (36 ± 2.1%, and 38 ± 8.8%, respectively).^65^

Navidpour *et al.* synthesized a new series of niflumic acid derivatives 8–11 according to the reported procedures.^67–70^ All the synthesized compounds were tested for their anti-inflammatory activity using carrageenan induced rat paw edema.^63^ Compound 8 was the most potent derivative (percentage of edema inhibition = 95.37 ± 4.45% at 30 min). Moreover, compound 8 was tested for its analgesic effect using rat writhing method,^71^ and showed percentage of inhibition = 49.43%. Compounds 9, 10 and 11 exhibited excellent anti-inflammatory activities (percentage of edema inhibition = 77.92 ± 4.04%, 79.07 ± 5.60%, 87.48 ± 5.04%, at 30 min., respectively) and considerable analgesic activities = 52.23%, 59.69%, and 97.27%, serially.^68^

In accordance, a new mefenamic acid analogues 12a, and 12b (Fig. 11) were successfully prepared through the nucleophilic attack of the 1,4-phenylenediamine on 2-chloronicotinic acid.^72^ Compounds 12a and 12b revealed unique analgesic and anti-inflammatory activities considering that celecoxib, indomethacin, diclofenac selectivity indices (SI) are 9.26, 0.39, and 1.19, successively as a standard reference. Furthermore, 12a and 12b showed the superior selective inhibition activity towards COX-2 enzyme. These compounds were also tested for their GIT ulcerogenic effect. The results for both COX-1 and COX-2 at 10 μM concentration were motivating towards their COX-2 enzyme selectivity. Compound 12a showed 66.9% of COX-1 inhibition and 77.2% of COX-2 inhibition, while compound 12b possessed 68.5% of COX-1 inhibition and 77.4% of COX-2 inhibition compared to celecoxib using 10 μM percentage of inhibition. Statistical analysis of the data was performed using one-way Anova followed by Tukey’ s Karmer post hoc test for multiple comparisons at probability levels of *p* < 0.05, results were considered statistically significant. Guided with the above promising results the gastric ulcerative effect was investigated and compared to the same three standard drugs. Upon recording the number of sores and their severity compound 12b was a competitor to the control with null sores while compound 12a exhibited 0.4 ± 0.01 sores with severity 0.74 ± 0.01%. On the other hand, celecoxib, diclofenac and indomethacin showed 3.3 ± 0.02, 4.1 ± 0.1 and 9.5 ± 0.4 sores with severity 7.1 ± 0.2, 8.6 ± 0.3 and 17.3 ± 0.5, respectively.^72^

### 2.2 1,3,4-Oxadiazole-containing compounds

**Fig. 12:**
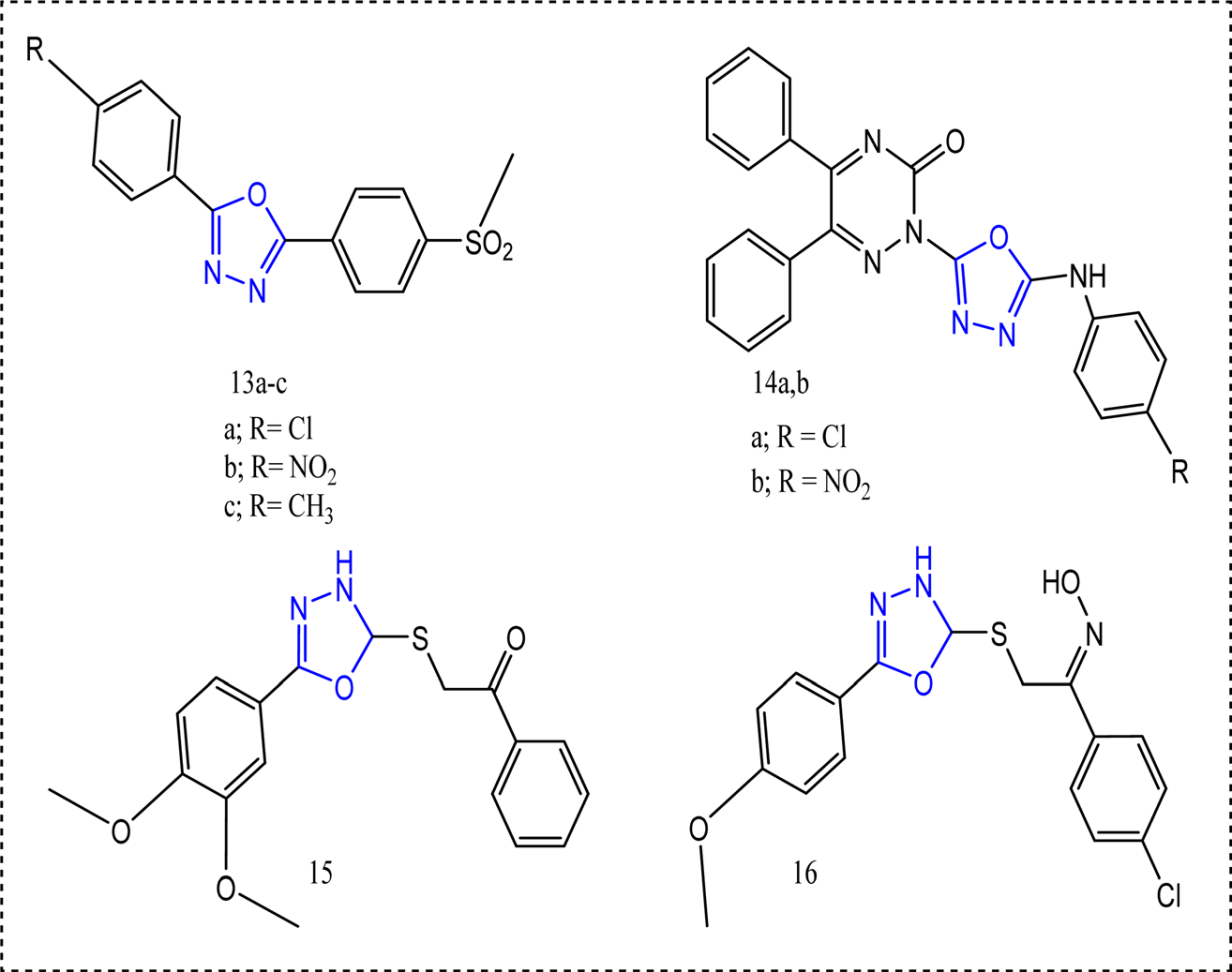
The most promising oxadiazole-containing compounds.

Grover's team built up the scaffold 2,5-diaryl-1,3,4-oxadiazoles derivatives 13a–c (Fig. 12).^73^ The successful procedure started with the reaction of the appropriate benzaldehydes with the substituted hydrazides to yield the resultant acyl hydrazones followed by the reaction with *N*-bromosuccinimide (NBS) and triethylamine. The oxidation of thiomethyl group using oxone as an oxidizing agent in acetonitrile/water finally afforded the sulfone functionality.^73^ Series 13 revealed a significant COX-2 selectivity. Compound 13a exhibited COX-2 selectivity index = 132.83 *c.f.* celecoxib selectivity index 379.80, and IC_50_ of COX-2 (0.74 μM). Methylsulfonyl group was a distinguishing pharmacophore, since the compounds bearing methylsulfonyl group showed higher docking scores against COX-2 (46.27–55.53 kcal mol^−1^) in comparison to thiomethyl derivatives (10.52–53.14 kcal mol^−1^).^73^

Applying the hybrid molecular technique^74^ Banerjee's team conjoined five-membered heterocyclic rings with diphenyltriazine-3-ones 14a,b.^75^ 5-Arylamino-1,3,4-oxadiazole compounds 14a, and 14b were prepared *via* the oxidative cyclization of thiosemicarbazides that results in elimination of H_2_S. Attaching electron-withdrawing group to 5-membered heterocyclic ring increased the activity significantly.^75^ Using carrageenan induced paw edema technique, it was obvious that compounds 14a,b had a quick onset (after 2 h) related to other compounds. In addition to the exceptional anti-inflammatory activities (60.97%, and 61.56%, respectively *c.f.* celecoxib 62.26%).^75^

**Fig. 13:**
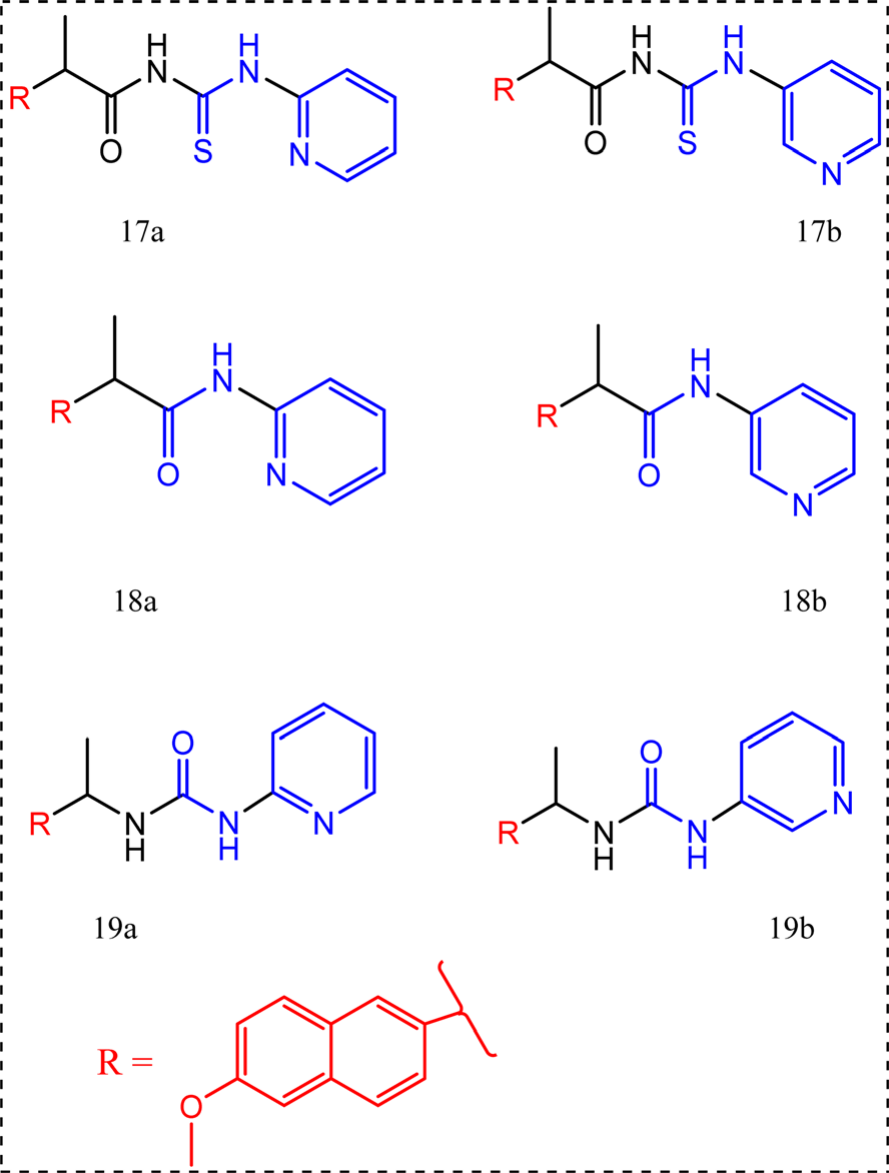
Pyridine-containing members.

In accordance, the 1,3,4-oxadiazole member 15 and the oxime core 16 exhibited noticeable anti-inflammatory activities displaying 72% and 83.33% inhibition of carrageenan induced edema,^63^ respectively. Nevertheless, both compounds 15 and 16 lacked a better selectivity profile towards COX-2 inhibition. The mono and dimethoxyphenyl substituted group seemed to increase the anti-inflammatory activity but not COX-2 selectivity. Compound 15 showed a noticeable inhibition of COX-1 IC_50_ = 1.10 ± 0.18 μM weighed against COX-2 IC_50_ = 2.30 ± 0.19 μM. On the other hand, 16 showed COX-1 IC_50_ 0.94 ± 0.10 μM and COX-2 IC_50_ 5.00 ± 0.61 μM^76^ (Fig. 13).

### 2.3 Pyridine and 2-pyridone-containing compounds

The pyridinamide functionality was a new attracting scaffold,^77^ the synthesis of these new scaffolds begins with reacting naproxen and thionyl chloride to prepare the corresponding acid chloride followed by reaction with 2-or 3-aminopyridine to yield the pyridinecarboxamides 17a,b. Moreover, compounds 18a,b were synthesized through reacting 2- or 3-aminopyridine with naproxenoyl isothiocyanate. Naproxen acid chloride was also converted to the urea derivatives 19a,b*via* the reaction with sodium azide, then addition of amino pyridines *via* the curtius rearrangement of the acid azide.^77^

All the synthesized compounds 17a,b, 18a,b, 19a,b were tested for their anti-inflammatory activity using carrageenan induced rat paw edema using naproxen with percentage of edema inhibition = 25.93% as a standard drug giving their percentage of edema inhibition (40.03%, 33.12%, 33.67%, 33.67%, 35.61%, and 33.12%, respectively). In another words their potency compared to potency of the standard drug naproxen after 5 h of induced inflammation were sequentially 1.54, 1.27, 1.29, 1.29, 1.37, and 1.27 times more than naproxen.^77^

**Fig. 14:**
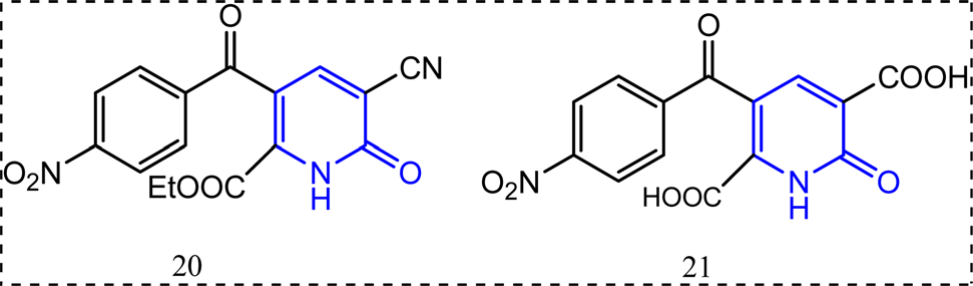
2-Pyridone-containing compounds.

Compounds 20 and 21 (Fig. 14) are examples for the 2-pyridone-containing compounds possessing anti-inflammatory activity.^78^ They exhibited percentage of edema inhibition (98.9%, and 90.8%) with 1.25 μg. Accordingly, from these results compound 20 exceeded indomethacin (98.2%) in its anti-inflammatory activity.^79^ Compound 20 was also identified as a selective COX-2 inhibitor, whereas 21 lacked COX-2 selectivity (Fig. 14).

### 2.4 Tetrahydropyrido[2,3-*d*] pyrimidine-containing compounds

**Fig. 15:**
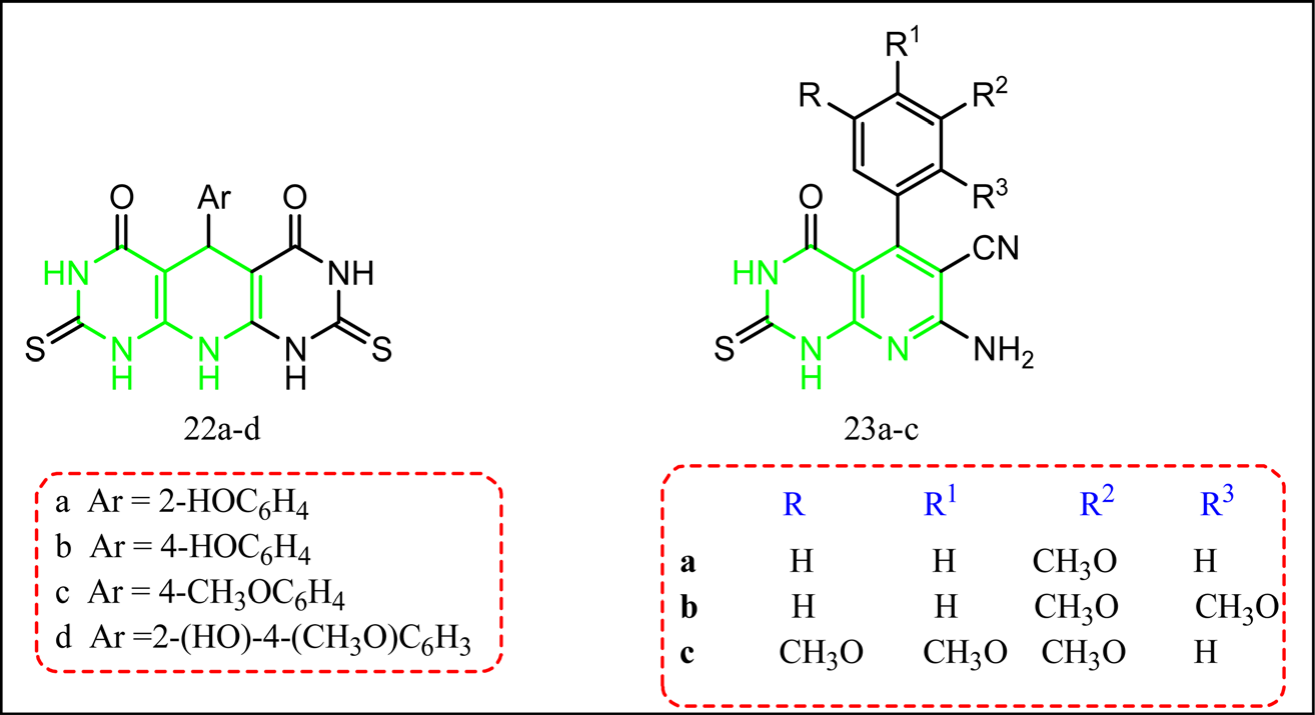
Tetrahydropyrido[2,3-*d*] pyrimidine-containing compounds.

Abdelgawad *et al.* reported some compounds with pyrido[2,3-*d*:6,5-*d*]dipyrimidine-4,5-diones 22a–d, and the tetrahydropyrido[2,3-*d*]pyrimidine-6-carbonitrile derivatives 23a–d (Fig. 15).^27^ All compounds were tested for their *in vitro* anti-inflammatory activity. This was achieved by comparing the IC_50_ of an enzyme immunoassay (EIA) kit for ovine as the distinguishing parameter,^80^ and *in vivo* anti-inflammatory activity using carrageenan induced rat paw edema model and celecoxib was the standard drug.^27,63^ Compounds 22b,d and 23c showed selective COX-2 inhibition as shown by their selectivity indices (SI = 4.99, 6.43, and 17.08, respectively) *c.f.* celecoxib COX-2 selectivity index 6.61. Moreover, compound 23c exhibited an edema inhibition profile better than the standard drug celecoxib's result (74%). The gastrointestinal ulcerogenicity was also evaluated for the target molecules 22d and 23c with ulcer index 2.25, and 1.5, sequentially *c.f.* indomethacin ulcer index = 22.5.^81,82^

### 2.5 Pyridazinone-containing compounds

**Fig. 16:**
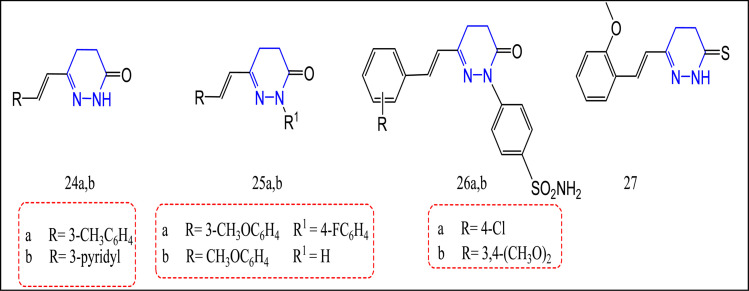
Pyridazinone/thione-containing compounds.

The synthesis of several novel pyridazinone-containing compounds as selective COX-2 inhibitors was reported.^83^ Biological evaluation revealed that compounds 24a,b, and 25a,b possess the most promising selective COX-2 inhibition. The COX-2 IC_50_ ranged between 15.56 and 19.77 nM. Moreover, their selectivity indices (SI) were 24, 38, 35, and 24, respectively. All synthesized compounds were 1.4 to 2.2 times more selective than celecoxib COX-2 IC_50_ 17.79 ± 0.69 nM, and (SI) 17.18. Moreover, the *in vivo* results through the carrageenan induced rat paw edema technique and ulcerogenicity profile revealed that compounds 24b, 25a,b displayed greater anti-inflammatory activity in comparison with celecoxib, and no ulcerogenic effect was recognized by any of these compounds^83^ (Fig. 16).

Recently, the pyridazinone derivatives 26a,b, and pyridinethione one 27 were prepared and tested for their selective cyclooxygenase-2 inhibitory activities.^84^ Compounds 26a,b, and 27 exhibited significant influence towards COX-2 enzyme inhibition in comparison with celecoxib. COX-2 IC_50_ for these compounds were 67.23, 43.84, and 53.01 nM, respectively *i.e.*, 1.1–1.7-fold more effective than celecoxib and indomethacin with IC_50_ = 73.53, 739.2 nM, successively. Compound 26b possessed a superior COX-2 selective inhibition with a SI = 11 equivalent to indomethacin and celecoxib. Furthermore, compound 26b exceeded both celecoxib and indomethacin in its ulcerogenic profile^84^ (Fig. 16).

### 2.6 Benzopyran-containing compounds

**Fig. 17:**
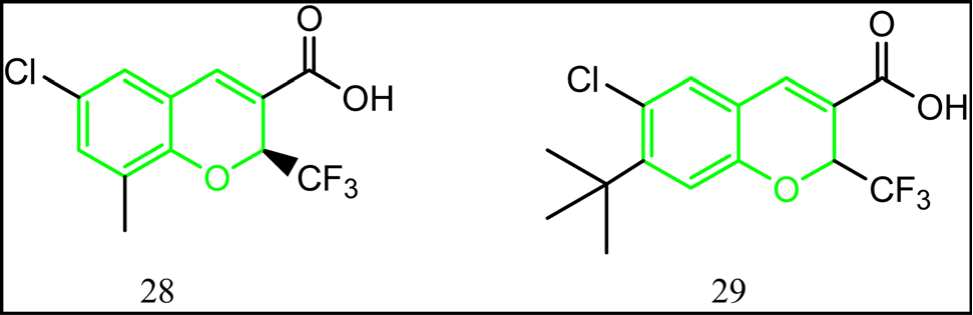
Benzopyran-containing compounds.

From its name, benzopyran is a bicyclic system composed of benzene ring fused to the six-membered hetero ring pyran. Chromene is the retained name of this class.^85^ Novel benzopyran derivatives were synthesized according to the reported procedure (Fig. 17).^86^ All the synthesized compounds were tested for their COX-1/COX-2 inhibitory ability. Compound 28 was considered the lead compound of this class. The selectivity index for 28 IC_50_ of COX-1/IC_50_ of COX-2 was 69 folds with excellent anti-inflammatory activity. The structure activity relationship study (SAR) revealed that substitution at position 7 is beneficial for both selectivity and enhanced activity.^86^ In accordance, compound 29 exhibited 16.45 folds selectivity towards COX-2 (IC_50_ = 0.062 μM).^86^

### 2.7 Pyrrolizine-containing compounds

**Fig. 18:**
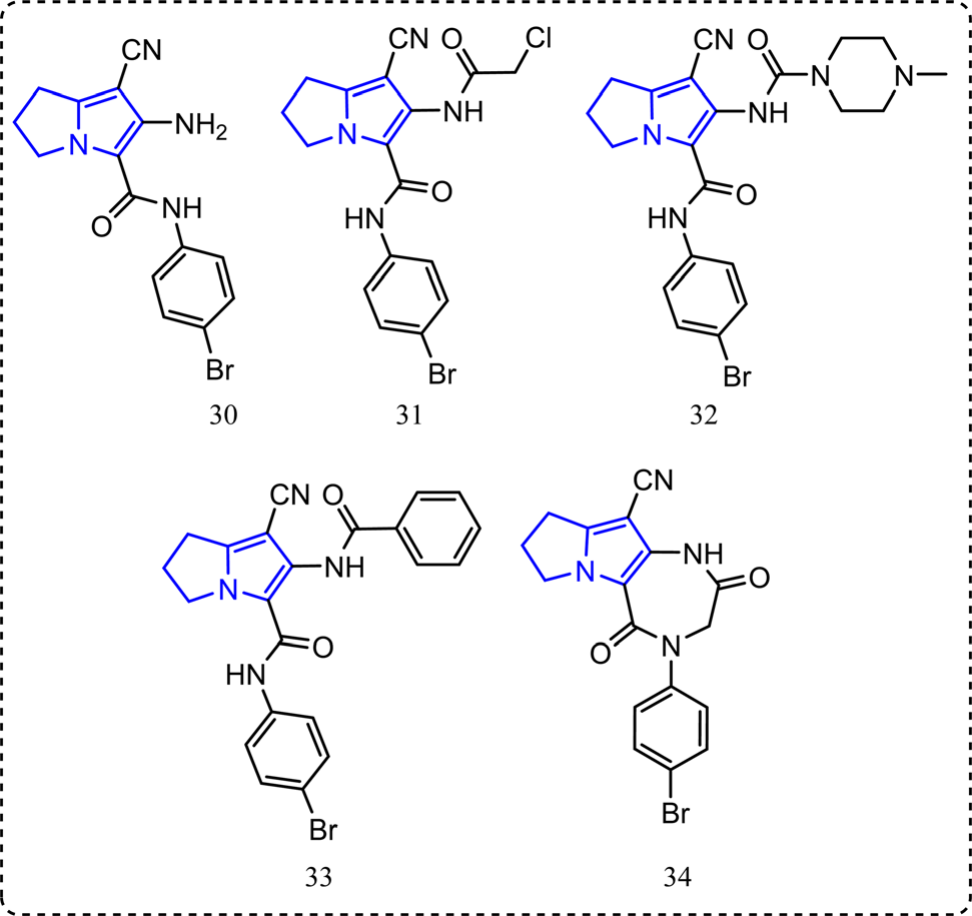
Pyrrolizine-containing compounds.

Recently, a series of pyrrolizine derivatives were synthesized achieving greater anti-inflammatory activity with minimal side effects.^87^ All the synthesized compounds were *in vitro* tested for their COX-1/COX-2 inhibitory ability using a COX colorimetric inhibitor screening assay kit.^88^ Also, their *in vivo* anti-inflammatory activities were applied using carrageenan induced rat paw edema.^63,87^ Compounds 30–34 (Fig. 18) revealed an exceptional anti-inflammatory activity and acceptable selectivity towards COX-2 inhibitory activity. Accordingly, compounds 30, 31, 32, 33 and 34 (Fig. 18) showed IC_50_ of COX-1/IC_50_ of COX-2 equal to 3.64, 3.48, 3.21, 3.17, and 2.89 folds, respectively *c.f.* 0.02 for indomethacin, while their percentages of inhibition of edema thickness after 3 hours were (44.79%, 52.31%, 16.37%, 24.91%, and 50.58%, sequentially) compared to ibuprofen 40.82%. Percentage of inhibition of ulcer formation was (66.38%, 68.18%, 66.38%, 80.66%, and 83.69%, respectively *c.f.* zero protection for ibuprofen).^87^

### 2.8 Isonicotinic acid-containing compounds

**Fig. 19:**
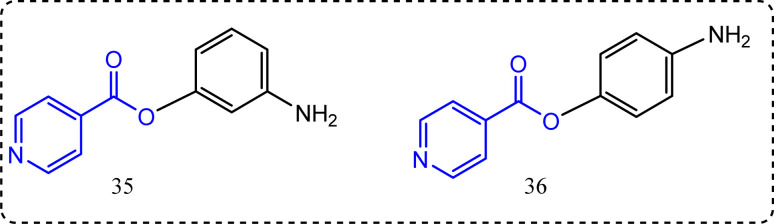
Isonicotinic acid-containing compounds.

New scaffolds of isonicotinic acid containing derivatives were reported by Zaheer *et al.*^89^ The anti-inflammatory activity was evaluated *via* chemiluminescence technique.^90^ The results revealed that compounds 35 and 36 (Fig. 19) showed superior anti-inflammatory activity, with percentage inhibition = 95.9%, and 67.3%, respectively. Compounds 35 and 36 showed COX-2 IC_50_ = 1.42 ± 0.1, and 8.6 ± 0.5 excelling IC_50_ of ibuprofen itself (11.2 ± 1.9) as a standard drug.

### 2.9 Pyrazoles and pyrazolo[3,4-*b*]pyridines-containing compounds

**Fig. 20:**
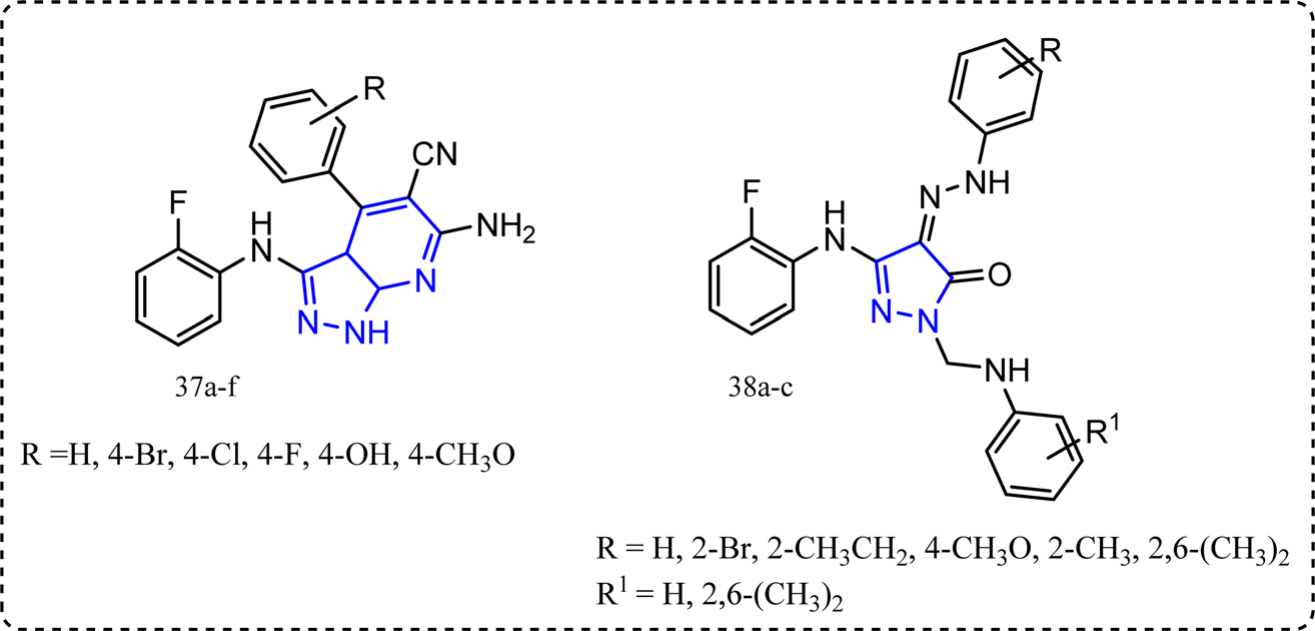
Pyrazoles and pyrazolo[3,4-*b*]pyridines-containing compounds.

Mohamed *et al.* synthesized novel pyrazoles and pyrazolo[3,4-*b*]pyridines derivatives 37a–f and 38a–c (Fig. 20).^91^ All the target molecules were tested for their *in vitro* anti-inflammatory activity using cayman COX (ovine/human) inhibitor assay using diclofenac sodium,^92^ indomethacin and celecoxib as a standard drug. Pyridines derivatives 37a–f SI (IC_50_ COX-1/IC_50_ COX-2) ranged between 16.346 to 104.878 folds compared to celecoxib with SI 308.163, while compounds 38a–c exhibited SI = 258.333, 297.917, and 267.391, respectively. Furthermore, all the target molecules were evaluated for their *in vivo* anti-inflammatory activity using carrageenan induced rat paw edema. Compounds 38a–c exhibited *in vivo* anti-inflammatory activities 61%, 64%, and 62%, respectively *c.f.* 69% for celecoxib. Additionally, their ulcerogenic incidence was (20–30%).^93,94^

## 3. Conclusion

Commencing these facts, we tried to track the most updated trials to attain novel compounds with promising anti-inflammatory, analgesic activities and if possible higher COX-2 enzyme selectivity. To the best of our knowledge no perfect balanced anti-inflammatory with minimal side effects was discovered. Consequently, the continuous and active trials for the development of new and selective coxibs with diminished side effects are still a hot research spot.

New scaffolds that may be eye-catching to explore could be built upon the promising findings from previous studies,^95–97^ future work should further explore the anti-inflammatory potential of 2-substituted arylaminonicotinic acid derivatives bearing an aminosulfonyl moiety. This approach combines the nicotinic acid pharmacophore with the biologically active sulfonamide moiety to develop compounds with improved anti-inflammatory properties. Specifically, it would be beneficial to investigate a wider range of substituents at positions 2 and 4 of the phenyl ring to optimize the anti-inflammatory efficacy. Additionally, replacing the carboxylic acid moiety with a 2-aminothiadiazole ring may be explored. The choice of the thiadiazole ring as a substitute for the carboxyl group is hypothesized to enhance anti-inflammatory activity while minimizing gastrointestinal toxicity.

## Data availability

No primary research results, software or code have been included and no new data were generated or analyzed as part of this review.

## Conflicts of interest

There are no conflicts to declare.
